# Endoscopic treatment of an unusual post-sleeve gastrectomy complication: first use in clinical practice

**DOI:** 10.1055/a-2471-7918

**Published:** 2024-12-12

**Authors:** Ting Wei, Dan Liu, Qingfen Zheng, Muhan Li, Jinglong Lv, Bingrong Liu

**Affiliations:** 1 191599 Department of Gastroenterology and Hepatology, The First Affiliated Hospital of Zhengzhou University, Zhengzhou, China

Laparoscopic sleeve gastrectomy (LSG), a common form of bariatric surgery, can lead to complications, such as staple-line leaks and gastric stenosis 1 2 3 . Herein, we report a rare post-LSG complication and its successful endoscopic treatment ( Video 1 ). A 24-year-old man was admitted with poor glycemic control for 2 months. His medical history included metabolic syndrome, and his body mass index was 33.9 kg/m ^2^ . He had undergone LSG with placement of a gastric decompression tube. On his first postoperative day, 800 mL of cloudy yellow fluid was drained, and the patient experienced abdominal pain. Several pulls on the gastric decompression tube in an attempt to remove it failed.

We present an exceptionally rare post-sleeve gastrectomy complication and its successful endoscopic treatment.Video 1

Endoscopy revealed that the tip of the gastric decompression tube was caught at the cardiac anastomosis, anchored by staples ( Fig. 1 **a** ). Initial attempts to remove the staples using snares were unsuccessful because of their firm fixation. Foreign body forceps were then used to extract the staples, allowing uneventful removal of the gastric decompression tube. After its removal, a perforation (2 × 3 cm) was identified at the cardiac anastomosis ( Fig. 1 **b** ). Given the defect's size and friable mucosa, a purse-string suture technique was employed. A nylon loop attached to a transparent cap was introduced into the gastric lumen via the endoscope, while SureClips were deployed through the biopsy channel. The loop was anchored to the distal edge of the defect with the first clips ( Fig. 1 **c** ). It was then progressively secured with six additional clips, slowly tightening until the defect was fully closed( Fig. 1 **d, e** ). The perforation was successfully closed in 15 minutes.

**Fig. 1:**
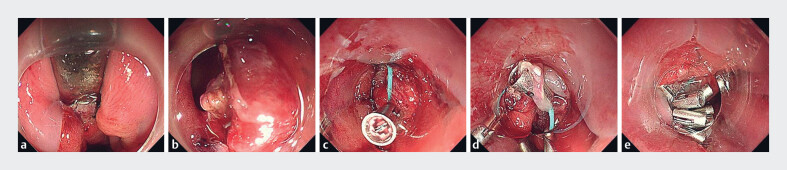
Endoscopic images showing: **a** a gastric decompression tube caught at the cardiac anastomosis, anchored by staples; **b** a perforation at the cardiac anastomosis, measuring 2 × 3 cm, following removal of the decompression tube; **c** a nylon loop fixed to the distal edge of the defect using clips; **d** further fixation of the loop around the defect, with six additional clips being placed; **e** complete closure of the defect following placement of the loop and clips.

A new gastric decompression tube was placed, which remained patent postoperatively. Three days post-procedure, an upper gastrointestinal contrast examination showed no leakage ( Fig. 2 ), and the patient was discharged 14 days later.

**Fig. 2:**
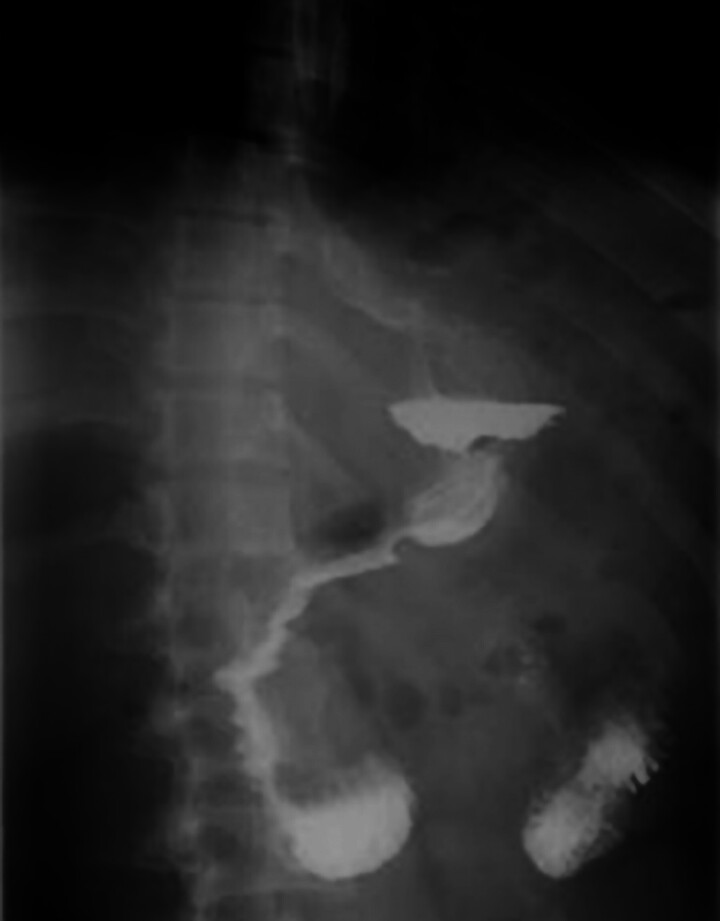
Upper gastrointestinal contrast examination showing no signs of leakage 3 days after the procedure.

Gastric decompression tube retention is an exceptionally rare post-LSG complication. Surgeons must remain vigilant during intraoperative handling and postoperative removal. If this complication is suspected, endoscopy is an effective intervention.

Endoscopy_UCTN_Code_TTT_1AO_2AI

## Notes

Endoscopy E-Videos https://eref.thieme.de/e-videos
*E-Videos* is an open access online section of the journal *Endoscopy* , reporting on interesting cases and new techniques in gastroenterological endoscopy. All papers include a high-quality video and are published with a Creative Commons CC-BY license. Endoscopy E-Videos qualify for HINARI discounts and waivers and eligibility is automatically checked during the submission process. We grant 100% waivers to articles whose corresponding authors are based in Group A countries and 50% waivers to those who are based in Group B countries as classified by Research4Life (see: https://www.research4life.org/access/eligibility/ ). This section has its own submission website at https://mc.manuscriptcentral.com/e-videos .
